# Physician Workforce Response to the COVID-19 Pandemic at an Academic Medical Center

**DOI:** 10.1017/dmp.2020.377

**Published:** 2020-10-12

**Authors:** Laurie G. Jacobs, Jason A. Korcak, Marygrace Zetkulic

**Affiliations:** Department of Medicine, Hackensack Meridian School of Medicine, Nutley, New Jersey; Hackensack University Medical Center, Hackensack, New Jersey

**Keywords:** disaster planning, physician workforce, redeployment

## Abstract

**Objectives:**

The aim of this study was to describe the planning, implementation, and outcome of an acute care physician supplemental workforce during the local coronavirus disease 2019 (COVID-19) surge at a 771-bed academic medical center, from March 25 to May 5, 2020, in New Jersey, United States.

**Methods:**

The Department of Medicine sought participation by “independent” and redeployed “employed” physicians to provide acute hospital care, as well as assistance with occupational health and family communication. Plans addressed training, compensation, clinical privileges, malpractice, and collaboration with the existing hospitalist service.

**Results:**

Redeployed employed physicians (81% internists) selected either acute care (*n* = 68; median age, 52 y [range, 32-72 y]; 28% female) or non-face-to-face supportive roles (*n* = 69; median age, 52 y [range, 32-84 y]; 28% female). The redeployed physician group totaled 474 twelve-h daytime shifts typically caring for 10 patients per day. Six employed physicians refused redeployment, and only 3 independent physicians participated (all acute care). Of note, COVID-19 infection occurred in 10 hospitalists and intensivists, and in several redeployed physicians.

**Conclusions:**

Successful physician workforce staffing for medical disasters, such as the COVID-19 pandemic, requires consideration of personal risk, as well as medicolegal, financial, and clinical competency issues.

The onset of the coronavirus disease 2019 (COVID-19) pandemic forced hospitals to nimbly respond to an unprecedented increase in hospital volume and acuity by mobilizing an acute care medical workforce to provide safe and high-quality care. Prior disasters have required emergency and surgical services.^1^ To improve efficiency and quality, many United States (US) hospitals have employed physicians in inpatient and outpatient settings who practice alongside “independent” (or “voluntary”) physicians. Although most physicians feel an obligation to provide urgent care during a disaster,^2^ they may no longer simply volunteer as in the past,^3^ as they often consider this responsibility of employed physicians, in addition medicolegal issues and clinical preparedness^4-6^ concerns.

On March 2, 2020, COVID-19 arrived at our 771-bed academic medical center, part of a state-wide network and medical school in New Jersey, United States. Within weeks, it was a pandemic epicenter under a state-of-emergency executive order. Bed capacity expanded to 935, including intensive care unit (ICU) beds (48 to 153) and a 72-bed unit with reverse airflow, rapidly constructed in the former cafeteria. Pediatric admissions dwindled to less than 10, nonurgent surgery ceased under a state mandate, and ambulatory care evaporated in favor of newly reimbursable telemedicine.^7^ Updates to our emergency operations plan, developed during Hurricane Sandy (2012), did not address the physician workforce. It was clear that the anticipated surge of COVID-19 patients would rapidly outstrip the usual 300-inpatient capacity of the Department of Medicine’s independent internists and employed hospitalists.

We present the Department of Medicine’s physician workforce and operational plan and implementation during the March 25 to May 5, 2020, COVID-19 local surge. This descriptive study received exempt status from our Internal Review Board.

## METHODS

The Department leadership (Chairperson, Vice Chairperson, Internal Medicine [IM] Residency Program Director) developed a plan to recruit a “supplemental” physician workforce, to the hospitalists and independent physicians, who regularly provide inpatient care. Although many physicians have hospital credentials, with or without admitting privileges (Table 1), to meet US insurance requirements, most rely on hospitalists to provide inpatient care. This “supplemental” workforce was drawn from this pool of credentialed physicians, as well as from outside the institution.

**TABLE 1:** Department of Medicine Physicians by Employment Status and Participation in the Supplemental Physician Services

Department of Medicine Physicians			Employed				Independent		
			*Redeployment*		*No Redeployment*				
*Subspecialty*	Grand Total^a^	Admitting Privilege Total^b^	*Acute care*^c^	*Nonface-to-face Care*^d^	*Excluded*^e^	*Refusal*^f^	*Supple-mental*^g^	*No Supple-mental*	*No Admitting Privilege*^h^
Allergy	4	2	2	0	0	0	0	0	2
Cardiology	154	147	33	35	0	0	1	78	7
Critical Care	14	14	0	0	13	0	0	1	0
Dermatology	18	13	0	2	0	0	0	11	5
Endocrinology	16	13	3	0	0	0	0	10	3
Gastroenterology	48	29	2	0	0	1	0	26	19
Gen Internal Med	137	124	18	22	0	2	1	81	13
Geriatrics	14	13	3	7	0	0	0	3	1
Heme-Oncology	28	28	0	0	7	0	0	21	0
Hospital Med	35	35	0	0	34	1	0	0	0
Infectious Dis	7	7	0	0	4	0	0	3	0
Nephrology	26	23	0	0	10	0	0	13	3
Palliative Care	3	3	2	0	1	0	0	0	0
Pulmonary	21	18	4	1	4	1	1	7	3
Rheumatology	11	7	1	2	0	0	0	4	4
Total	536	476	68	69	72	5	3	258	60

a All credentialed physicians, with and without admitting privileges.

b All credentialed physicians with admitting privileges, employed and independent.

c Employed physicians redeployed to acute and critical care (3 cardiologists, 2 pulmonologists).

d Non–face-to-face care: occupational health telemedicine or Family Communication Service.

e Employed physicians excluded from redeployment: critical care, hematology-oncology, hospitalists, infectious disease, nephrology, pulmonary.

f Employed physicians who refused redeployment.

g Participants in the Supplemental Physician Workforce.

h Physicians with hospital credentials (for insurance purposes) without admitting privileges.

Daily webinars, email, telephone, and electronic surveys were used for physician recruitment as in-person meetings were suspended to slow viral spread. Communications included epidemiologic, treatment, research, and regulatory updates to increase physician comfort with treating COVID-19. Although internists were targeted initially, recruitment quickly expanded to include all credentialed physicians. These efforts were further consolidated in a network command center that provided daily Web-based and email updates, and assisted with procurements and staffing, within 2 wk. Several principles were established: (1) All physicians would be compensated for their activity. (2) All physicians would be credentialed with malpractice coverage for acute medical care. (3) Independent physicians would be encouraged to participate. (4) All employed physicians would be subject to redeployment as a principle of fairness. (5) Redeployment to inpatient care would be encouraged without considering age, gender, or personal circumstances; however, requests for non–face-to-face service (occupational health telemedicine or the new family communication team) would be accepted *a priori.* (6) Inpatient service consisted of 3 to 4 consecutive 12-h daytime shifts with a 10-patient census located on the same unit. Assignments were based on other clinical practice obligations and hospital volume.

Independent physicians were offered short-term contracts with a standard shift rate and were responsible for their own malpractice coverage and professional charges. During emergency operations, our Medical Staff bylaws provide credentialed physicians “emergency privileges” for care beyond their usual scope of practice, and “disaster privileges” for nonaffiliated US-licensed physicians. While temporary malpractice coverage was provided if unavailable, a statewide executive order waived liability for pandemic COVID-19 care.

The Department requested redeployment of employed internists from ambulatory practice to the supplemental workforce (acute or non–face-to-face care) for some, or all, of their contracted work hours, with network leadership support. Intensivists, hospitalists, infectious disease, nephrology, and oncology consultants were excluded due to concurrent demand. Employed neurologists, urologists, and pediatricians also agreed to redeployment. Professional collections (or work relative value units) were applied to each physician’s practice. Malpractice was covered. Work beyond contracted hours was compensated at a standard rate, irrespective of specialty. Temporary housing in a hotel was available.

Required training sessions included: (1) in-person and/or video training for use of personal protective equipment, offered hourly; (2) electronic medical record refresher course, and; (3) 1-h clinical training by Departmental leadership to review treatment guidelines, conduct of care, clinical trials, and resources. A network-wide interdisciplinary team developed COVID-19 clinical guidelines, updated daily online, discussed respiratory care, laboratory monitoring, therapeutics, and clinical trials. Occupational health and family communication teams had their own training.

Supplemental physicians assigned to inpatient care worked in partnership with the hospitalist team. Hospitalists performed all new COVID-19 admissions to ensure quality and safety of the initial treatment plan, then redistributed patients to supplemental physicians or hospitalists based on geographic location. Hospitalists also provided on-demand clinical assistance, overnight coverage, trainee supervision, and continued staffing of the rapid response and cardiac arrest teams. Physician’s supplemental shifts were staggered, allowing real-time staffing changes based on volume. Redeployed providers with intensivist training were asked to assist medical ICU teams. A “Discharge Team” of nurse practitioners with care transition experience was deployed to assist with discharge planning, medication reconciliation, and communication with families navigating the complex transition for COVID-19 patients.

Using the Accreditation Council for Graduate Medical Education’s existing Extraordinary Circumstances Policy, residents and fellows were re-assigned to medical or critical care services, under supervision. During the pandemic, our designated institutional official attested to Stage 3 Pandemic Emergency Status, allowing the IM Residency Program Director to assume responsibility for educating and redeploying trainees from other subspecialties and disciplines with their program director’s consent. Inpatient student rotations were suspended.

## RESULTS

Despite the many disruptions due to the pandemic, this plan provided structure for our physician workforce. The supplemental physicians (104) who provided acute care (Table 1) included 71 internists, 7 neurologists, 19 employed pediatricians (who provided non-COVID care), 2 plastic surgeons, and 5 urologists. Thirteen unaffiliated physicians participated with disaster privileges. Surgeons provided a central and arterial “line placement service.” Orthopedists assisted nurses to place patients in a prone position to enhance oxygenation.

**FIGURE 1:**
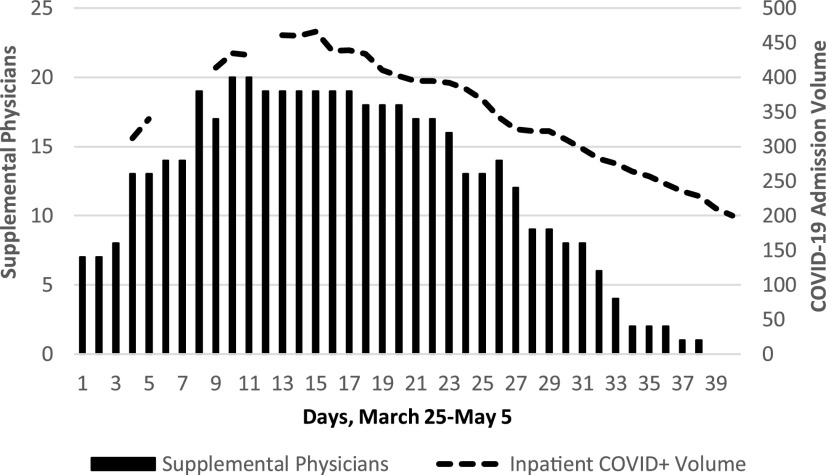
Supplemental Physician Staffing and COVID-19 Admission Volume by Day.

Only 3 of 321 independent internists (median age, 54 y [range, 31-82 y]; 13% female), participated, 2 in critical care. The age and gender of redeployed internists providing acute care (*n* = 68; median age, 52 y [range, 32-72 y]; 28% female) or non–face-to-face care (*n* = 69; median age, 52 y [range, 32-84 y]; 28% female), did not differ. Those physicians who were excluded from redeployment (n = 72, median age, 43 y [range, 32-71 y]; 38% female) included hospitalists, intensivists, and consultants. Several pregnant physicians elected to serve in either role; a pregnant hospitalist requested a leave of absence after several weeks. Staffing (81% by internists) was successfully matched to admission volume, ranging from 2 to 20 supplemental physicians daily (Figure 1), providing 474 shifts in total.

Qualitative unsolicited comments about redeployment were positive. Physicians appreciated the opportunity to contribute in a meaningful way and enhance their clinical competence. They also valued the Departmental support and training as some had not provided acute care since residency. Those performing family communication described this experience very positively; occupational health was difficult. Five employed physicians refused redeployment or to come in to work due to concerns about infection risk and accepted an unpaid leave of several weeks. Three returned; 2 resigned.

There were no reports of major quality or safety issues. Several redeployed physicians developed COVID-19 infection. Two were admitted as inpatients; a third physician who worked in the ICU, tragically died from presumed COVID-19–related illness. Additionally, 5 hospitalists and 6 intensivists contracted COVID-19. One had a prolonged ICU admission, and another, disabling symptoms.

The IM residents (40) and fellows (5) were reassigned to the ICU. Communication skills observed by supervising attendings regarding goals of care, risks and benefits of interventions, and delivery of bad news rapidly strengthened. One urology and 1 obstetrics resident assisted in inpatient care. Dental residents (16) assisted with clinical trial enrollment and postdischarge phone calls.

## DISCUSSION

The creation of a successful physician workforce plan to meet the needs of the COVID-19 pandemic required a highly structured program using all available physician resources. In addition to existing hospitalists and intensivists, the supplemental workforce consisted primarily of redeployed employed providers in lieu of independent physicians. The majority of independent physicians no longer admit patients in the inpatient setting, and as a result, were not positioned to offer inpatient assistance during the pandemic, and had an expectation that employed physicians would initially fill the need. The smaller group of independent physicians already significantly engaged in inpatient care experienced a surge in their own volume. Many independent physicians also admit to other local hospitals, preventing them from participating in 12-h shifts. Other barriers to their participation included a pivot toward telemedicine, apprehension about infection risk, and liability concerns despite the state waiver. Midway through the 6 wk, several independent physicians expressed interest in participating as their typical outpatient volume remained low.^8^ By this time, the local pandemic was estimated to be near peak, and the schedule had been filled with redeployed physicians who did not incur additional institutional expense. Many of these issues may reflect the idiosyncrasies of today’s US health system.

Employed physicians selecting acute or non–face-to-face redeployment did not differ in age or gender. Other factors influenced their choice, notably, the risk of acquiring COVID-19. Ultimately, the redeployed physicians’ infection rate was low. Hospitalists, intensivists, and residents, sustained more exposure, particularly during intubations and other sustained interactions. Some chose acute care due to professional responsibility. As outpatient volume fell, and telemedicine adoption was initially slow, inpatient care allowed employed physicians to generate contractually incentivized professional billing, which was unavailable for occupational medicine or family calls.

## CONCLUSIONS

Departments of Medicine play a pivotal role in disaster preparedness physician workforce planning. Our experience highlights the need to address all factors motivating physicians’ participation, including infection fears, financial drivers, and existing relationships. Physician staffing during this pandemic underscores the complexities of the US physician workforce, including employed versus independent practice models and a tendency toward specialization. Our experience demonstrates that these barriers can be rapidly overcome by active leadership, frequent communication, sensitivity to training and operations, and leverage of a flexible, engaged, and compassionate medical staff. While there is evidence that physician volunteerism rates in a prolonged medical disaster are low,^9,10^ our plan supplemented physician altruism with a pragmatic and supportive approach.
